# Radiological and Laboratory Features of Multiple Sclerosis Patients With Immunosuppressive Therapy: A Multicenter Retrospective Study in Japan

**DOI:** 10.3389/fneur.2021.749406

**Published:** 2021-10-13

**Authors:** Shinji Ashida, Hirofumi Ochi, Mio Hamatani, Chihiro Fujii, Ryusei Nishigori, Kazuyuki Kawamura, Sadayuki Matsumoto, Masanori Nakagawa, Ryosuke Takahashi, Toshiki Mizuno, Takayuki Kondo

**Affiliations:** ^1^Department of Neurology, Graduate School of Medical Science, Kyoto Prefectural University of Medicine, Kyoto, Japan; ^2^Department of Neurology and Geriatric Medicine, Ehime University Graduate School of Medicine, Ehime, Japan; ^3^Department of Neurology, Kyoto University Graduate School of Medicine, Kyoto, Japan; ^4^Department of Neurology, National Hospital Organization Minami Kyoto Hospital, Kyoto, Japan; ^5^Department of Neurology, Kitano Hospital, Osaka, Japan; ^6^Department of Neurology, North Medical Center Kyoto Prefectural University of Medicine, Kyoto, Japan; ^7^Department of Neurology, Kansai Medical University Medical Center, Osaka, Japan

**Keywords:** multiple sclerosis, treatment, MRI, immunosuppressant, serum autoantibodies

## Abstract

**Background:** Multiple sclerosis (MS) is a relapsing, inflammatory, and demyelinating disease of central nervous system showing marked clinical heterogeneity. Many factors might influence the choice of relapse prevention drug, and treatment response varies among patients. Despite the enlargement of disease-modifying drugs for MS (MS-DMDs), some patients have been treated with corticosteroid and/or immunosuppressant (CS/IS).

**Objective:** To clarify the radiological and laboratory features of MS treated with CS/IS for relapse prevention.

**Methods:** Clinical records including radiological and laboratory findings, and drugs used for relapse prevention were reviewed retrospectively.

**Results:** Out of 92 consecutive MS patients, 25 (27%) were treated with CS/IS. The followings were observed less frequently in patients treated with CS/IS than in those with MS-DMDs: three or more periventricular lesions, ovoid lesions, subcortical lesions, typical contrast-enhancing lesions, negative for serum autoantibodies, and positive for oligoclonal bands in the cerebrospinal fluid. Multiple logistic regression analysis revealed that the absence of typical contrast-enhancing lesions and positivity for serum autoantibodies were independent factors associated with CS/IS prescription (odds ratio 25.027 and 14.537, respectively).

**Conclusion:** In this cohort of Japanese patients clinically diagnosed with MS, radiological and serological findings atypical of MS were observed more frequently in patients treated with CS/IS than in those with MS-DMDs as a part of MS therapy. The absence of contrast-enhancing lesions typical of MS and positivity for serum autoantibodies were independent factors strongly associated with CS/IS use.

## Introduction

Multiple sclerosis (MS) is a chronic immune-mediated inflammatory disease of the central nervous system (CNS) associated with demyelination and axonal damage (1). The heterogeneity of the disease pathogenesis and clinical course poses a challenge regarding diagnosis and patient management. Although the differential diagnosis of MS involves multiple diseases, neuromyelitis optica spectrum disorder (NMOSD) is a major differential diagnosis. Differentiation between MS and NMOSD has an important implication regarding patient management because patients respond differently to treatment, and some disease-modifying drugs for MS (MS-DMDs) are ineffective or exacerbate NMOSD (2). The identification of autoantibodies, such as anti-aquaporin-4 (AQP4) antibody (3), and anti-myelin oligodendrocyte glycoprotein (MOG) antibody (4, 5), greatly aids in the differentiation of NMOSD from MS. However, some patients remain seronegative despite having a clinical phenotype consistent with NMOSD. Differential diagnosis is particularly difficult in these seronegative patients who are clinically borderline between MS and NMOSD, although there have been reports of clinical and paraclinical discriminators of the two diseases (6–8). Indeed, there is marked disagreement even among experts on the diagnosis of MS/NMOSD-overlap patients, while the level of agreement regarding the treatment of these patients is marked (9). Experts recommend immunosuppression as first-choice treatment for most MS/NMOSD-overlap patients, and also some MS patients (9). This suggests that immunosuppressants are prescribed for some patients as a part of MS therapy. However, studies of paraclinical characteristics of patients who have received immunosuppressants remain insufficient. To clarify the most important clinical factors related to corticosteroid and/or immunosuppressant (CS/IS) use, we compared the radiological and laboratory findings between MS patients treated with CS/IS and MS-DMDs.

## Methods

### Patients and Study Design

This was a retrospective study conducted in three MS-referral hospitals in the Kansai region of Japan: Kyoto Prefectural University of Medicine (Kyoto, Japan), Kyoto University Hospital (Kyoto, Japan), and Kitano Hospital (Osaka, Japan). Patients' demographic information, and radiological and laboratory findings at the time of diagnosis were reviewed and their association with prescription drugs for relapse prevention was analyzed. Therapeutic strategies were decided under the supervisions of MS specialists in each hospital. MS-DMDs included interferon-beta, glatiramer acetate, fingolimod, dimethyl fumarate, and natalizumab. Immunosuppressants included azathioprine, tacrolimus, cyclosporine, methotrexate, and cyclophosphamide. For brain MRI analysis, we focused on three features: ovoid lesions, subcortical U-fiber lesions, and 3 or more periventricular lesions (PL ≥ 3), which may be useful to differentiate MS from NMOSD (6, 8, 10). We also evaluated patterns of contrast-enhancement on brain MRI. For laboratory analysis, positivities of serum anti-nuclear antibody (>1:640 dilutions), anti-Ro/SS-A antibody, anti-La/SS-B antibody, and anti-thyroid antibodies were examined. If cerebrospinal fluid (CSF) analysis was performed, information on oligoclonal bands (OCB) positivity, cell counts, and protein concentrations was also collected.

### Ethics

This study was approved by the Medical Ethics Committee of Kyoto Prefectural University of Medicine according to the tenets of the Declaration of Helsinki. Using an opt-out approach, we provided information on the research, including the purpose, and guaranteed that patients could request exclusion.

### Statistical Analysis

Statistical analysis was performed by using JMP® 13 (SAS Institute Inc., Cary, NC, USA). A *p* < 0.05 was considered significant. Age, disease duration, and the duration of treatment were dealt as continuous variables and analyzed by using Mann-Whitney *U* test. χ^2^ test was used for testing relationships on categorical variables. The multiple logistic regression was used to analyze factors associated with CS/IS use.

## Results

### Demographic Characteristics

**Figure 1:**
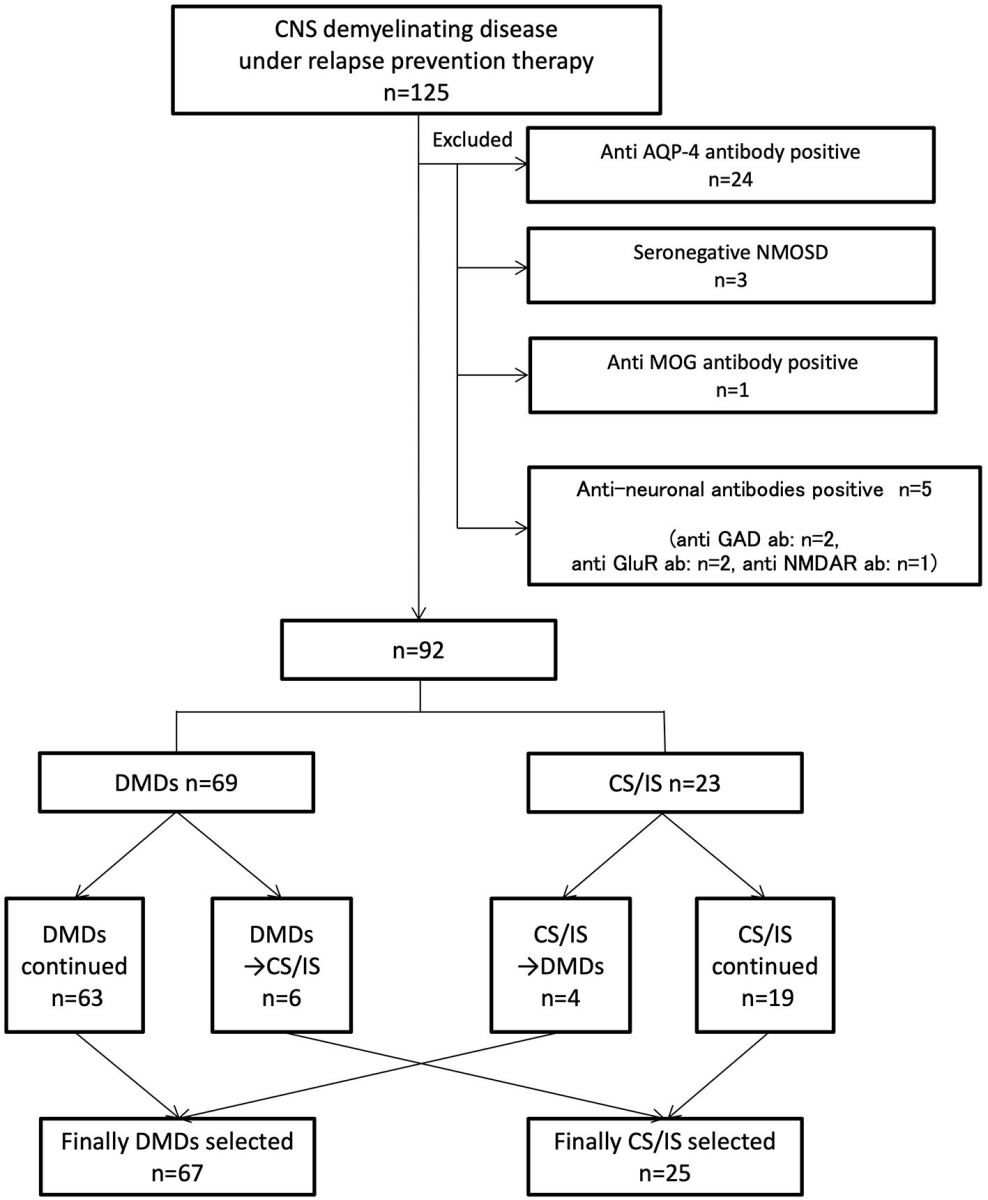
Indicates inclusion/exclusion criteria and grouping of patients. A total of 125 consecutive patients with inflammatory demyelinating central nervous system diseases receiving relapse prevention therapy were included. Thirty-three patients were diagnosed with potential explanations other than MS. Ninety-two patients were divided into MD-DMDs and CS/IS groups depending on the treatment choice. CNS, central nervous system. MS, multiple sclerosis. AQP-4, aquaporin-4. NMOSD, neuromyelitis optica spectrum disorder. Ab, antibody. MOG, myelin oligodendrocyte protein. GAD, glutamic acid decarboxylase. GluR, glutamate receptor. NMDAR, N-methyl-D-aspartate receptor. DMDs, disease-modifying drugs. CS/IS, corticosteroid and/or immunosuppressant.

A total of 125 consecutive patients with relapsing inflammatory diseases of the CNS receiving relapse-preventive therapy from October 2016 to March 2017 were included in this study. After careful exclusion of potential explanations other than MS through clinical and paraclinical evaluations, 92 consecutive patients were finally included. Of the 125 patients, 33 were excluded from the analysis: 24 were seropositive for AQP4 antibody by the cell-based assay, three fulfilled seronegative NMOSD criteria (11), one was seropositive for MOG antibody by the cell-based assay, and five were seropositive for other anti-neuronal antibodies by the cell-based assay (two was seropositive for anti-glutamate receptor antibody, one was seropositive for anti-Glutamic Acid Decarboxylase antibody, and one was seropositive for N-methyl-D-aspartate receptor antibody) (Figure 1). The identified 92 consecutive patients were diagnosed with MS and satisfied the McDonald 2010 criteria for the diagnosis of MS (12). Among the identified 92 patients, 66 (72%) were tested for anti-AQP4 antibody, and 6 (6.5%) for anti-MOG antibody. All of the tested patients were seronegative for anti-AQP4 and anti-MOG antibody. Of the 92 patients, 67 were female (73%), and 25 were male (27%). The mean age at onset was 33.0 ± 10.5 years; mean age at enrollment was 44.7 ± 10.9 years; and mean treatment duration was 7.2 ± 4.3 years.

The median Expand Disability Status Scale (EDSS) was 2.0 [0–6.5]. The median relapse number was 2.5 [0–12].

### Therapeutic Strategies for Relapse Prevention

69 patients (75%) had their initiation of treatment with MS-DMDs: interferon-beta in 61, fingolimod in six, dimethyl fumarate in one, and natalizumab in one. Six of the 69 patients (8.7%) were switched to corticosteroid and/or immunosuppressant (CS/IS) thereafter. The reasons for switching included disease exacerbation after the initiation of MS-DMDs (*n* = 3), insufficient efficacy (*n* = 1), and serious side effect (*n* = 1). One patient was later proven to have concomitant collagen disease. Among the 63 patients who continued to receive MS-DMDs, 28 patients were switched to other MS-DMDs because of insufficient efficacy (*n* = 25) or side effects (*n* = 3).

23 patients (25%) had their initiation of treatment with CS/IS. No patient showed exacerbation after the initiation of CS/IS. Nineteen of the 23 patients (83%) continued to receive CS/IS. Four patients (17%) were switched to MS-DMDs because of insufficient efficacy: two were switched to dimethyl fumarate, one to fingolimod, and one to natalizumab. One of them showed a reduction in the annual relapse rate from 3.0 to 1.5 and the others remained relapse-free after switching to MS-DMDs.

### Radiological and Laboratory Features Are Associated With Prescription Drugs for Relapse Prevention

Finally, 25 patients (27%) received CS/IS and 67 patients (73%) received MS-DMDs for long-term maintenance therapy (Figure 1; Table 1). The duration of currently ongoing treatment was 4.5 ± 0.3 years, and there was no difference between MS-DMDs and CS/IS groups (4.5 ± 0.6 years vs. 4.6 ± 0.4, respectively, *p* = 0.67) (Table 1). To clarify the clinical factors related to CS/IS use for relapse prevention, we first compared the type of first clinical manifestation between patients finally treated with CS/IS and those with MS-DMDs (Table 1). Regarding the type of the first clinical manifestation, visual disturbance was more frequent in patients treated with CS/IS than those with MS-DMDs, with marginal significance (64 vs. 42%, respectively, *p* = 0.04).

**Table 1:** Demographic and clinical manifestation of patients treated with MS-DMDs and CS/IS.

	**CS/IS**	**MS-DMDs**	***p*-value**
	**(*n* = 25)**	**(*n* = 67)**	
Demographic characteristics
Age	49.3 ± 12.9	43.4 ± 10.0	0.08
Gender (Female)	20 (80%)	46 (70%)	0.5
Duration of current treatment	4.6 ± 0.4	4.5 ± 0.6	0.67
EDSS	2.0 [0–5]	2.5 [0–6.5]	0.06
Type of first clinical manifestation
Motor	3 (12%)	15 (22%)	0.25
Sensory	3 (12%)	13 (19%)	0.39
Visual	16 (64%)	28 (42%)	**0.04**
Spinal cord	3 (12%)	10 (15%)	0.7
Cerebellar	1(4%)	2 (3%)	0.82

*MS, multiple sclerosis; DMD, disease-modifying drug; CS/IS, corticosteroid and/or immunosuppressant; EDSS, The median of Expand Disability Status Scale. p-value below 0.05 are shown in bold*.

We next compared lesion distribution on MRI and MRI features between patients finally treated with CS/IS and those with MS-DMDs (Table 2). Regarding lesion distribution on MRI, the involvement of subcortical, cerebellum and spinal cord was significantly higher in patients treated with MS-DMDs than those with CS/IS (42 vs. 16%, *p* = 0.02, 16 vs. 0%, *p* = 0.03 and 66 vs. 36%, *p* = 0.01, respectively). PL ≥ 3 and ovoid lesions were significantly more common in patients treated with MS-DMDs than those with CS/IS, respectively (87 vs. 36%, *p* < 0.0001, and 93 vs. 52%, *p* < 0.0001, respectively). Contrast-enhancing lesions were significantly more common in patients treated with MS-DMDs than those with CS/IS (73 vs. 28%, respectively, *p* < 0.0001). The rates of lesions with nodular and/or open-ring/ring enhancement patterns was higher in patients treated with MS-DMDs than those with CS/IS (98 vs. 43%, *p* = 0.0005).

**Table 2:** Radiological and laboratory features of patients treated with MS-DMDs and CS/IS.

	**CS/IS**	**MS-DMDs**	***p*-value**
	**(*n* = 25)**	**(*n* = 67)**	
Lesion distribution on MRI			
Subcortical lesions	4 (16%)	28 (42%)	**0.02**
Cerebellum	0	11 (16%)	**0.03**
Brainstem	12 (48%)	25 (37%)	0.77
Spinal cord	9 (36%)	44 (66%)	**0.01**
MRI features			
PV ≥3	9 (36%)	58 (87%)	** <0.0001**
Ovoid lesions	13 (52%)	62 (93%)	** <0.0001**
CE lesions	7 (28%)	49 (73%)	** <0.0001**
Nodular/open ring/ring CE lesions	3/7 (43%)	48/49 (98%)	**0.0005**
Laboratory findings			
Positive for serum autoantibodies	6 (24%)	2 (3%)	**0.0016**
anti-nuclear antibody (>1:640 dilutions)	0/23 (0%)	1/56 (2%)	0.73
anti-Ro/SS-A antibody	4/22 (18%)	0/53 (0%)	**0.0015**
anti-Ro/SS-B antibody	1/22 (5%)	0/53 (0%)	0.29
anti-thyroid antibodies	3/13 (23%)	1/23(4%)	0.13
Presence of CSF OCB	2/20 (10%)	20/47 (43%)	**0.02**
CSF cell count >50/μL	1/21 (5%)	1/46 (2%)	0.3
CSF protein level >100mg/dL	1/21 (5%)	1/46 (2%)	0.3

*MS, multiple sclerosis; DMD, disease-modifying drug; CS/IS, corticosteroid and/or immunosuppressant; MRI, magnetic resonance imaging; PV, periventricular lesions; CE, contrast-enhancing; CSF, cerebrospinal fluid; OCB, oligoclonal bands. p-value below 0.05 are shown in bold*.

We further compared the laboratory findings between patients finally treated CS/IS and those with MS-DMDs. Six of the 25 patients treated with CS/IS were seropositive for autoantibodies: two was seropositive for Ro/SS-A antibody, two for anti-thyroid antibodies, one for both Ro/SS-A and Ro/SS-B antibodies, and one for both Ro/SS-A and anti-thyroid antibodies. While only two of the 67 patients treated with MS-DMDs were seropositive for autoantibodies: one was seropositive for serum anti-nuclear antibody and one for anti-thyroid antibodies. The positive rate of serum autoantibodies was lower in patients treated with MS-DMDs than those with CS/IS (3 vs. 24%, respectively, *p* = 0.0016). OCB positivity was higher in patients treated with MS-DMDs than those with CS/IS (43 vs. 10%, respectively, *p* = 0.02).

We finally performed multiple logistic regression analysis of radiological and laboratory findings between patients treated with MS-DMDs and those with CS/IS (Table 3). OCB positivity was excluded from this analysis since this test was not conducted for all patients. The multiple logistic regression analysis revealed that the absence of nodular/open ring/ring contrast-enhancing lesions [odds ratio = 25.027 (1.559–401.821), *p* = 0.02] and positivity for serum autoantibodies [odds ratio = 14.537 (1.809–116.814), *p* = 0.01] were independent factors strongly associated with CS/IS use based on currently ongoing therapy. Patients without nodular/open ring/ring contrast-enhancing lesions showed the highest odds of CS/IS use.

**Table 3:** The multiple logistic regression analysis for the factors associated with CS/IS use.

**Based on currently ongoing maintenance therapy**	**Standardized partial regression coefficient**	**Odds ratio**	**95% confidence interval**	***p*-value**
Absence of ovoid lesions	1.365	3.917	0.663-23.141	0.13
PV <3	0.875	2.4	0.389–14.793	0.34
Absence of CE lesions	−1.47	0.23	0.018–2.870	0.25
Absence of nodular/open ring/ring CE lesions	3.22	25.027	1.559–401.821	**0.02**
Positive for serum autoantibodies	2.677	14.537	1.809–116.814	**0.01**

*MRI, magnetic resonance imaging; PV, periventricular lesions; CE, contrast-enhancing; CS/IS, corticosteroid and/or immunosuppressant; CI, confidence interval. p-value below 0.05 are shown in bold*.

### Clinical, Radiological, and Laboratory Features of Patients Who Were Switched Their Treatment

Six of the 69 patients initially treated with MS-DMDs (8.7%) were switched to CS/IS. All of the six switchers were seronegative for anti-AQP4 antibody. Two switchers showed seizure or consciousness disturbance as their first clinical presentation, but the others showed clinical manifestations typical of demyelination. Three switchers showed PV ≥ 3 and the others did not. Five switchers showed contrast-enhancing brain lesions without typical features of MS, and the other did not show any contrast-enhancing brain lesion. Two switchers were seropositive for Ro/SS-A antibody. Two of the six patients switched to CS/IS showed positivity for OCB.

Four of the 23 patients initially treated with CS/IS (17%) were switched to MS-DMDs. Their clinical manifestations were typical of demyelination and none of the switchers were seropositive for autoantibodies. All of the four patients showed PL ≥ 3, ovoid lesions, subcortical lesions, contrast-enhancing brain lesions typical of MS. Two of the four patients switched to MS-DMDs showed positivity for OCB.

## Discussion

In this study, radiological and serological features were associated with the therapeutic choice in patients with an established diagnosis of MS, after the exclusion of any alternative diagnosis and meeting MRI criteria for MS (12). A total of 27% of our MS patients were finally treated with CS/IS, and the absence of typical contrast-enhancing lesions and positivity for serum autoantibodies were independent factors associated with CS/IS use.

Since the initial inclusion of MRI in the diagnostic work-up for MS in 2001 (13), it has become the main procedure of choice for corroborating a clinical MS diagnosis. MRI criteria for MS rely in the detection of white matter lesions in the CNS, showing typical lesion morphology and locations of MS lesions. Thus, it serves to help clinical neurologists not only evaluate MS dissemination in space and time but also exclude other conditions that can mimic MS. However, not all MS patients show typical MRI findings, and around 10% of NMOSD patients show MS-like MRI findings (14–16). Some patients share features of both MS and NMOSD, and so might be difficult to definitively diagnose. This raises another important clinical issue regarding the treatment of MS patients where there is a radiological or laboratory feature atypical of MS but a suspicion of an alternative diagnosis is not clinically informative. In this study, we found that PL ≥ 3, ovoid lesions, subcortical lesions, nodular and/or open-ring/ring contrast-enhancing lesions, negative for serum autoantibodies, and positive for oligoclonal bands were observed less frequently in patients treated with CS/IS, all of which are known to be useful findings to discriminate MS from other neurological diseases in the CNS (6–8, 17). In other words, patients without radiological, and serological findings typical of MS have more frequently used CS/IS for relapse prevention. In addition, we found for the first time that the absence of contrast-enhancing lesions typical of MS and positivity for serum autoantibodies were strongly associated with more frequent use of CS/IS. Interestingly, although the numbers are limited (*n* = 6), none of the patients switched from MS-DMDs to CS/IS showed contrast-enhancing brain lesions typical of MS, and two of the six patients (33%) were seropositive for autoantibodies.

In this study, 27% of MS patients were treated with CS/IS. In a previous international questionnaire-based study on the use of IS including azathioprine, cyclophosphamide, methotrexate, and mitoxantrone, around 10% of MS patients were treated with one of the four drugs (18). The frequency of IS use differed widely between countries: France had the highest use rate of 32.5%, with 12.7% in Italy, but it was rarely prescribed in some countries. In a subsequent study in Italy, 31.6% of MS patients were treated with IS, in which patients with an older age at therapy assignment, higher EDSS, and progressive disease course were more frequently treated with IS (19). The frequency of IS use was similar to our result, but we could not observe such clinical features in MS patients treated with CS/IS. The differences in MS-DMDs used and study patients might account for the discrepant results. Only interferon-beta and glatiramer acetate were used as MS-DMDs in the Italian study, while our study included dimethyl fumarate, fingolimod, and natalizumab.

This study had several limitations. First, it was limited by the small sample size. Second, it had a retrospective design and anti-AQP4 and anti-MOG antibodies were not measured in all of the identified 92 MS patients. Anti-AQP4 antibody was tested in 66 patients (72%): 44 (66%) in MS-DMDs group and 22 (88%) in CS/IS group. Only six patients (6.5%) were tested for anti-MOG antibody: 2 (3%) in MS-DMDs group and 4 (16%) in CS/IS group, because measurement of anti-MOG antibody was not covered by insurance in Japan. Considering that MOG antibody-associated disease (MOGAD), NMOSD and MS may share clinical, radiological and laboratory features (20), there is a possibility that patients with MOGAD or NMOSD were involved in this study, especially in the CS/IS group. Third, how and when to use CS/IS was based on the primary physicians' judgements. However, a mean treatment duration of over 4 years (4.6 ± 0.4 years in the MS-DMD group and 4.5 ± 0.6 years in the CS/IS group) suggests that relapse prevention drugs currently in use are appropriate or at least not harmful for patients.

In conclusion, radiological and laboratory findings were associated with therapeutic choice in patients clinically diagnosed with MS. The absence of contrast-enhancing lesions typical of MS and positivity for serum autoantibodies were independent factors strongly associated with CS/IS use. Further prospective clinical study with an appropriate design is needed to clarify the therapeutic efficacy of CS/IS in MS patients with radiological and serological features atypical of MS.

## Data Availability Statement

The raw data supporting the conclusions of this article will be made available by the authors, without undue reservation.

## Author Contributions

SA: designed this study, analyzed clinical and radiological data, and contributed to writing the manuscript. MH, CF, and RN: assisted in the study design, data interpretation, and collected patients' clinical data. KK: assisted in the study design and data interpretation. RT: assisted in writing the manuscript. HO, SM, MN, RT, and TM: assisted in data interpretation and writing the manuscript. TK: assisted and oversaw the study design, data interpretation, and supervised the process of writing the manuscript. All authors contributed to the article and approved the submitted version.

## Funding

This study was partially supported by Grant-in Aid for Scientific Research (C) (Grant Numbers JP 18K07528 and 21K07463) (HO) and Grant-in-Aid for Research Activity start-up (Grant Number JP 20K22786) (SA).

## Conflict of Interest

HO is on the scientific advisory board for Biogen Japan Ltd., Novartis Pharma KK, and Alexion Pharmaceuticals Inc.; received speaker honoraria from Bayer Yakuhin Ltd., Novartis Pharma KK., Mitsubishi Tanabe Pharma Corp., Alexion Pharmaceuticals Inc., Chugai Pharmaceutical Co., Ltd., Nihon Pharmaceutical Co., Ltd., and Takeda Pharmaceutical Co., Ltd. The remaining authors declare that the research was conducted in the absence of any commercial or financial relationships that could be construed as a potential conflict of interest.

## Publisher's Note

All claims expressed in this article are solely those of the authors and do not necessarily represent those of their affiliated organizations, or those of the publisher, the editors and the reviewers. Any product that may be evaluated in this article, or claim that may be made by its manufacturer, is not guaranteed or endorsed by the publisher.

## Abbreviations

MS: multiple sclerosis
NMOSD: neuromyelitis optica spectrum disorder
CS/IS: corticosteroid and/or immunosuppressant.
